# Dietary Fibre Intervention for Gut Microbiota, Sleep, and Mental Health in Adults with Irritable Bowel Syndrome: A Scoping Review

**DOI:** 10.3390/nu13072159

**Published:** 2021-06-23

**Authors:** Ran Yan, Lesley Andrew, Evania Marlow, Kanita Kunaratnam, Amanda Devine, Ian C. Dunican, Claus T. Christophersen

**Affiliations:** 1 School of Medical and Health Sciences, Edith Cowan University, Joondalup Drive, Perth 6027, Australia; l.andrew@ecu.edu.au (L.A.); e.marlow@ecu.edu.au (E.M.); k.kunaratnam@ecu.edu.au (K.K.); a.devine@ecu.edu.au (A.D.); i.dunican@ecu.edu.au (I.C.D.); c.christophersen@ecu.edu.au (C.T.C.); 2 Institute for Nutrition Research, Edith Cowan University, Joondalup Drive, Perth 6027, Australia; 3 WA Human Microbiome Collaboration Centre, School of Molecular and Life Sciences, Curtin University, Kent Street, Perth 6102, Australia; 4 Integrative Metabolomics and Computational Biology Centre, Edith Cowan University, Joondalup Drive, Perth 6027, Australia

**Keywords:** IBS, FODMAP, dietary fibre, gut microbiota, sleep, mental health, short-chain fatty acid

## Abstract

Irritable bowel syndrome (IBS) is a common functional gastrointestinal disorder affecting 4–5% of the global population. This disorder is associated with gut microbiota, diet, sleep, and mental health. This scoping review therefore aims to map existing research that has administrated fibre-related dietary intervention to IBS individuals and reported outcomes on at least two of the three following themes: gut microbiota, sleep, and mental health. Five digital databases were searched to identify and select papers as per the inclusion and exclusion criteria. Five articles were included in the assessment, where none reported on all three themes or the combination of gut microbiota and sleep. Two studies identified alterations in gut microbiota and mental health with fibre supplementation. The other three studies reported on mental health and sleep outcomes using subjective questionnaires. IBS-related research lacks system biology-type studies targeting gut microbiota, sleep, and mental health in patients undergoing diet intervention. Further IBS research is required to explore how human gut microbiota functions (such as short-chain fatty acids) in sleep and mental health, following the implementation of dietary pattern alteration or component supplementation. Additionally, the application of objective sleep assessments is required in order to detect sleep change with more accuracy and less bias.

## 1. Introduction

### 1.1. Irritable Bowel Syndrome

Irritable bowel syndrome (IBS), a common functional gastrointestinal disorder, is characterised by recurrent abdominal pain and alterations in bowel habits that include the coexistence of bloating, flatulence, and abdominal distention [1]. According to the symptom-based Rome IV diagnostic criteria, IBS can be subtyped into four categories: constipation dominant (IBS-C), diarrhoea dominant (IBS-D), mixed IBS (IBS-M), and un-subtyped (IBS-U) [2]. Globally, IBS had previously been estimated to affect 11–12% of the population [3], where this figure was corrected to 4–5% following the introduction of the Rome IV criteria in 2016 [4,5], such as 4.7% of adults in the United States, 4.6% in the United Kingdom, 4.5% in Canada [4]. According to a population-based cross-sectional survey, 7.9 % of Australian adults have a self-reported medical diagnosis of IBS [6]. Even though the aetiology of IBS remains unclear, emerging evidence suggests that IBS may be one of the disorders of gut-brain interaction [7,8], engaging homeostasis regulation via the gut-brain-microbiome axis [9,10]. Research to date suggests that 44% of IBS patients have associated mental health disorders, including depression and anxiety [11,12], where 37.6% of IBS patients have reported sleep problems, such as sleep fragmentation, poor sleep quality or reduced sleep duration [13,14,15]. There is also evidence to suggest and support that IBS is related to gut dysbiosis (unbalanced microbiota that lack microbial diversity and temporal instability [16]), leading to subtype-specific and symptom-relevant alterations in gut microbiota [17].

A survey of United States (U.S.) gastroenterologists has reported that 85% perceived a diet low in fermentable oligosaccharides, disaccharides, monosaccharides, and polyols (FODMAP) to be very/somewhat effective as a dietary therapy, where roughly three out of four IBS patients were consequently recommended to implement a low-FODMAP diet [18]. The survey also found that more than 50% of IBS patients always (13.8%)/usually (38.8%) intended to manage their IBS symptoms by themselves before seeking advice from a gastroenterologist [18]. The American College of Gastroenterology suggests a low-FODMAP diet as a means of improving global symptoms in people with IBS [19].

### 1.2. The Low-FODMAP Diet

A low-FODMAP diet (LFD) is an effective way to reduce gut symptoms in people with IBS [20,21,22]. Technically, the diet is made up of three phases: elimination/restriction (2–6 weeks), reintroduction, and personalisation, which help patients to target personal trigger foods, to identify individual tolerance levels, and to self-manage their symptoms in their daily life [23,24]. The effectiveness (self-reportedly adequate symptom control) of a 3/4-week LFD intervention can be up to 68–81% [22,25]. Similarly, in an Irish cohort study, 66% (86/127), 72% (53/74), and 76% (31/41) of patients reported being satisfied with the overall symptomatic improvement of an LFD at follow-up stages of 3, 6, and 12 months, respectively [26]. Accordingly, only 11% (14/127) of participants were willing to be re-introduced to high FODMAP foods at the 3-month follow-up because of their fear of recurrence of symptoms, where 81.1% (*n* = 60/74) and 70.7% (*n* = 29/41) continued the exclusion/restricted LFD at 6-month and 12-month follow-up, respectively [26].

Gastrointestinal dysbiosis has been confirmed as a characteristic of IBS, where *Lactobacillus* and *Bifidobacterium* are deficient in people with IBS when compared to healthy populations [27]. An LFD or reduced FODMAP intake can reduce bifidobacteria but does not necessarily normalise the dysbiotic gut environment [28,29]. A 4-week LFD has been shown to lead to an increased dysbiosis in 42% of people with IBS, and 46% had no change in dysbiosis after the 4-week LFD [30]. A recent systematic review has demonstrated that restriction of FODMAP intake, either in healthy subjects or patients with intestinal diseases, including IBS, can induce microbial alteration associated with dysbiosis, compared to prebiotic supplementation [28]. However, the microbial signature related to IBS symptom severity was not found to be associated with intake of FODMAP but rather negatively associated with microbial richness [31].

When combined, despite the symptomatic improvement of an LFD in the majority of IBS patients, further research is still required to examine the long-term effects on gut health as many IBS sufferers are reluctant to re-introduce trigger foods. If patients do not manage to replace high FODMAP foods with suitable low-FODMAP alternatives, they may also be reducing fibre intake simultaneously [23,32]. Therefore, in this scoping review, the low-FODMAP diet is regarded as a dietary fibre-related intervention.

### 1.3. Current Guidelines of Fibre Use in People with IBS

Modification of (not simply increasing or decreasing) fibre intake is one of the general dietary messages from NICE guidelines for dietary management in those with IBS since certain types of dietary fibres are not well tolerated, such as wheat bran [33]. Moreover, the dietary adjustment requires consideration of patients’ subtype, symptoms profile, and individual triggers.

Australian dietary guidelines currently recommend a suggested dietary target (SDT) daily fibre intake of 38 g for men and 28 g for women in order to reduce chronic disease risk, where adequate intake (AI) values for male adults is set at 30 g and for female adults at 25 g [34]. In a randomised controlled trial (RCT) of LFD intervention, only 6 (5%) IBS participants habitual intake achieved the U.K. national recommendation (30 g/d) [35]. In a French adult cohort, both healthy controls (*n* = 34,578) and people with IBS (*n* = 1870) were found to consume lower fibre intake than recommended (25 g/day), at mean levels of 19.4 g and 19.3 g, respectively [36]. A lower fibre intake can be problematic; healthy individuals who switched from a high-fibre diet to a low-fibre, high-sugar diet decreased their microbial diversity and increased permeability in their small intestine [37]. Increased permeability and impaired epithelial barrier function are often observed in the small and large bowel of IBS patients, especially in IBS-D [8,38,39].

Many reviews have elaborated on the types and characteristics of dietary fibre as well as mechanisms of action and benefits [40]. Fibre can come from natural food, such as vegetables, fruits, legumes, and nuts, as well as in supplement form where specific health benefits are related to different fibre types. Some fibres that are well tolerated by IBS individuals include psyllium, linseeds, oat bran [41,42]. Due to individual heterogeneity, IBS patients should consider their own tolerability, the fibre amount, and preparation (e.g., whole/ground seeds) when using different fibres to improve or manage symptoms. For instance, patients need to ensure adequate water consumption when adding psyllium to their food due to its soluble and viscous characteristics.

### 1.4. The Relationship of Dietary Fibre, SCFA, Sleep, Mental Health, and the Gut Microbiome

The functionality of dietary fibres in the human gastrointestinal tract is determined by their physiochemical properties, such as solubility, viscosity, and fermentability [42]. Moreover, the amount and type of fibre residue escaping small intestinal digestion and reaching the colon drives the extent of fermentation [43]. For example, the location of gut microbial fermentation of psyllium, with its soluble and low fermentable characteristics, occurs along the length of the colon to produce SCFA, whereas the fermentation of resistant starch (RS) occurs more proximally in the colon due to its higher fermentability and low solubility (or insolubility depends on the types of RS) [41]. Specific gut microbes tailor the degradation and fermentation of specific fermentable fibres [44]. Some foods are associated with a higher abundance of specific beneficial gut microbes; for example: a higher abundance of *Faecalibacterium prausnitzii* has been positively associated with a higher intake of fruits, red wine, and oily fish; whereas *Roseburia hominis* increased on a diet containing nuts, oily fish, vegetables, legume, cereals [45].

Specifically, *Faecalibacterium prausnitzii,* a commensal bacterium, is a suitable biomarker in certain gut conditions, such as inflammatory bowel disease [46], and may be a suitable candidate as a future probiotic [47]. *Faecalibacterium prausnitzii*-derived metabolites, such as butyrate, can restore the impaired intestinal barrier structure and function [48]. *Faecalibacterium prausnitzii* has been associated with IBS as well as other diseases and disorders, such as colorectal cancer, obesity, type 2 diabetes, non-alcoholic fatty liver disease, Alzheimer’s and Parkinson’s disease, major depressive disorder, and bipolar disorder [46]. In an observational case-control pilot study evaluating gut microbial composition in children with obstructive sleep apnoea syndrome (OSAS), the abundance of *Faecalibacterium prausnitzii* was reduced in the OSAS cohort, compared with the healthy subjects [49]. The genus of *Faecalibacterium* was also associated with reduced depressive symptoms and better sleep in patients with bipolar disorder [50]. Taking the specific microbe as an example can show the existence of intertwined associations among gut microbiota, sleep, and mental health. SCFAs are one of the pivotal links, though the whole mechanisms remain unclear.

SCFAs are generated from gut microbial fermentation throughout the colon [51], with SCFA levels declining along the large intestine because of the rapid uptake and metabolisation by colonocytes [52], with only an estimated 5% of bacteria-derived SCFA appearing in the stool [53]. Within all three major SCFAs components, butyrate is the most effective in the trophic properties and provides the primary fuel for the colonic epithelial cells to maintain their growth and integrity [54,55]. Butyrate contributes to maintaining host health with the anti-inflammatory and antioxidant features that affect the immune system [56] and prevent diseases such as colorectal cancer [57,58], diabetes, and obesity [59]. Butyrate concentration is mainly dependent on the quantity and quality of dietary fibre reaching the colon [60]. Research has shown higher concentrations of butyrate in human faeces to be associated with greater fibre intake [61]. Foods that are rich in dietary fibre, such as nuts, fruit, vegetable, and cereal, are also linked to a greater abundance of SCFA producers in the human gut microbiota [45]. Since a low-FODMAP diet may result in reducing intake of dietary fibre long term [62], and studies have shown that people following this diet have a lower production of butyrate in the faeces [63], the potential long-term influence of low-FODMAP diet/low intake of fibre among IBS population requires further research.

National data from a U.S. adults survey has quantitively demonstrated an association between the daily intake of total fibre and sleep duration, where <5 h sleep, 5–6 h sleep, and 9+ h sleep were associated with decreased intake at levels of 13.2 ± 10.1 g (mean ± standard deviation), 15.9 ± 10.9 g and 14.2 ± 8.7 g, respectively, whereas adults with normal sleep (7–8 h) had the highest intake at 16.6 ± 9.6 g [64]. As mentioned earlier, the LFD reduces the intake of fermentable fibres, which can lead to alterations of the gut microbiota and a reduction in fermentation in the large bowel, and as such can reduce the production of short-chain fatty acids (SCFA) that support colonic integrity and colonocytes growth [23,63,65,66,67]. Microbiota-derived SCFA has also been suggested to enhance sleep [68] and modulate host circadian clocks [69] in animal studies. This related to the circadian clock maintaining mammalian homeostasis and rhythmic physiology, such as the sleep-wake cycle, eating, and fasting [70]. Gut microbiome composition and SCFA is associated with sleep physiology, where microbial alterations relate to sleep problems [71,72,73], and also play a pivotal role in human mental health conditions [74,75,76,77,78]. A recent study has found that acute psychological stress can increase intestinal permeability in healthy volunteers [79], which can be decreased by probiotics and prebiotics [75]. Prebiotics and probiotics also have shown the capacity to improve mental health, including depression and anxiety [75], although the pathway and mechanism behind this remain unclear. Therefore, it is important to expand IBS-related research to cover all these areas together and to identify the relationships among them.

### 1.5. The Current Gap and the Purpose of This Scoping Review

**Figure 1:**
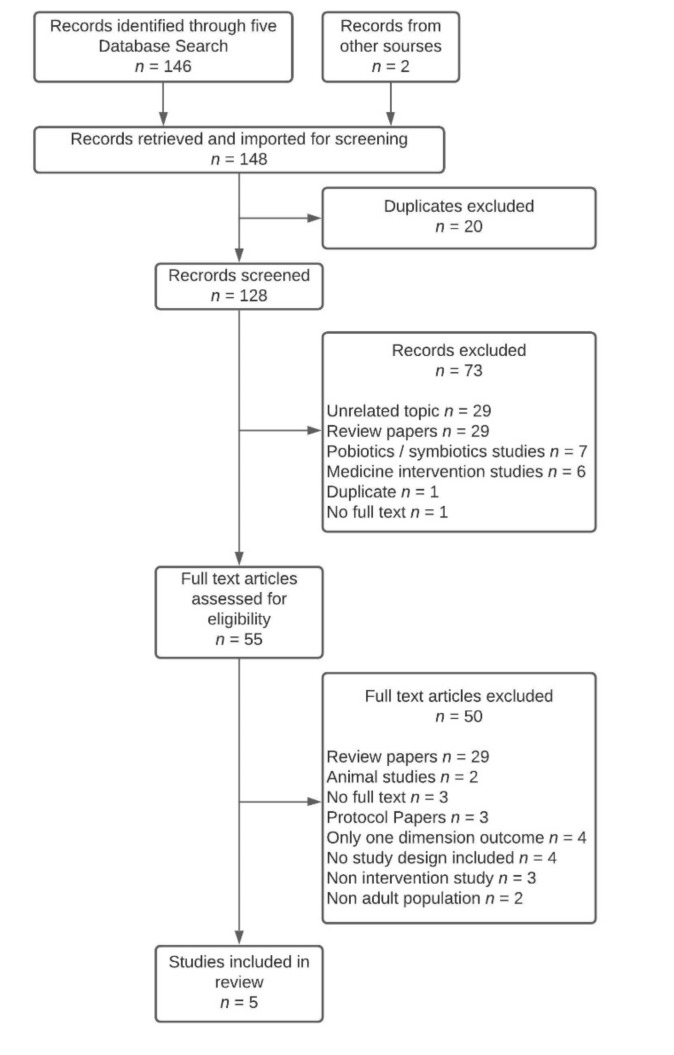
PRISMA flow chart of articles identification and inclusion.

IBS patients strive to manage their symptoms and normalise or improve their dysbiotic gut microbiota to experience better sleep and mental health, all bi-directionally linked to the gut microbiota (Figure 1). Emerging research based on healthy populations has focused on gut microbiota modulation via particular dietary fibre or specific prebiotics supplements [80,81,82]. These studies have identified promising and cost-effective approaches to improve human health. A few reviews recently published in 2020 have identified the associations among certain foods, nutrients, or diet intervention and their effects on sleep outcomes; however, they were all based on healthy adults [83,84]. However, among IBS populations, limited data are available. A few studies have focused on diet management or fibre supplementation. Their findings were specifically limited to gastrointestinal (GI) symptom improvement [42,85,86,87] rather than improvement in gut health, as well as in sleep and mental health. Accordingly, a gap remains in the research specific to IBS populations, particularly related to fibre-related intervention and its effects on the gut microbiota, sleep, and mental health. Hence, a scoping review that locates the existing evidence and identifies gaps in this area is essential to provide further direction regarding future explorations.

The aim of this scoping review is to map the research evidence that has provided IBS patients with dietary fibre-related intervention and described their effects on at least two of the following three outcomes: gut microbiota, sleep, or mental health.

## 2. Materials and Methods

### 2.1. Search Strategy

This review follows a scoping review protocol from the 2020 version of the Joanna Briggs Institute (JBI) manual [88], adhering to the Preferred Reporting Items for Systematic reviews and Meta-Analyses extension for Scoping Reviews (PRISMA-ScR) reporting standards [89]. A systematic search strategy was conducted to retrieve published research about fibre-relevant interventions in IBS patients recording outcomes on gut microbiota, sleep, and mental health. A literature search was performed on 26 February 2021, using five digital databases: MEDLINE, Embase, Web of Science, APA Psyc info, and CINAHL. The keywords used in the search are detailed in Table 1. Boolean operators (AND and OR), as well as the truncation, were used when each string was built for literature searching (Table 1). A complete set of terminology used in searching the literature combined at least four concepts with IBS and fibre as fixed concepts: IBS AND fibre AND gut microbiota AND sleep, IBS AND fibre AND gut microbiota AND mental health, IBS AND fibre AND sleep AND mental health, and IBS AND fibre AND gut microbiota AND sleep AND mental health. 

### 2.2. Selection Criteria

Articles were included if they were original research papers in IBS patients and had a fibre-related intervention and meet the inclusion criteria and did not meet the exclusion criteria, as detailed below.

Inclusion criteria:

1. The outcomes consisted of at least two out of three topics of: gut microbiota, sleep, and mental health.
2. Study types included peer-reviewed case-controls, cross-sectional studies, cohort studies, clinical trials, randomised controlled trials, non-randomised controlled trials, and pseudo-randomised trials.

Exclusion criteria:

(1) Non-human studies;
(2) Reviews, case reports, and systematic reviews;
(3) Subjects are non-adults;
(4) Non-English articles;
(5) Articles without full text or study design or results are not available;
(6) Interventions with probiotics, synbiotics, or medicine.

### 2.3. Study Selection

Records retrieved across the five searched databases were imported to the online reference management platform COVIDENCE [90]. After duplicates were automatically removed via COVIDENCE, records were screened using title and abstract, followed by a full-text article assessment against the inclusion and exclusion criteria by the first and second reviewers. The third and the fourth reviewers were consulted if the decision of any article remained disputed until a consensus was reached for all articles.

### 2.4. Data Extraction

Data were extracted and summarised from selected articles and transferred to a form with the headings: title, first author, country, year, type of study, sample size (N), as well as information of participants relating to mean age, age range, IBS subtypes, Rome diagnosis version, baseline fibre intake (g/day); interventions and group setting, and the outcomes in gut microbiota, sleep, and mental health as well as adverse effects. As the scoping review focused on a minimum of two out of three topics (gut microbiota, sleep, and mental health), only relevant outcomes were summarised and reported. Therefore, findings around other aspects such as quality of life and bowel symptoms were not extracted and reported.

## 3. Results

Based on the search strategy, 146 articles were selected from the databases, with two further articles from other sources, resulting in 148 records identified for screening via COVIDENCE. After the automatic removal of duplicates by COVIDENCE, 128 articles were scanned using titles and abstracts. During the first screening phase, 73 were excluded, leaving 55 articles to be assessed for eligibility in the second phase, where 50 articles were excluded (Figure 1). As a result, five articles were included in the scoping review (Table 2).

### 3.1. Characteristics of Included Studies

#### 3.1.1. Study Designs and Interventions

Of the five studies, three were randomised controlled trials, one was a prospective observational study, and one was a single-arm interventional study. Two studies added dietary supplementation to the habitual diets of participants, while the other three provided LFD intervention via dietitian or nutritionist consultation and instruction (Table 2).

#### 3.1.2. Setting and Participants Characteristics

The studies included in the scoping review were performed in the USA [91], France and Spain [92], U.K. [93], Australia and New Zealand [94], and Italy [95]. Four of the five studies were published within the last five years, three in 2017, one in 2019, and the remaining study in 2009. The Rome diagnosis version used was the current version at the time of the study. Only one study (published in 2009) used Rome II, while the other four used Rome III, which was released in 2006 and introduced the classification of four subtypes of IBS based on stool consistency [96].

The mean age of participants across the five studies was 43.9 years. The upper limit of age ranged from 60 to 79 across the five studies, where the lower limit was the same at 18 years except for one study where it was 16 years. One study limited participants to females with IBS-D, while the other four included both genders with all IBS subtypes.

#### 3.1.3. Dietary Fibre Intake Data

Two out of five studies reported data of dietary fibre intake, including baseline and treatment period. Bellini et al. implemented an 8-week LFD in IBS volunteers, and no statistical significance was found between the two time points, even though mean intake during LFD (17.5 ± 7.3 g) was lower than the one at baseline (20.5 ± 10.7 g/day). The other study using GOS as the treatment reported that the fibre intake remained unchanged among the groups (placebo group and prebiotic groups) during the course of the study. Eswaran et al. reported that nutrient intake was similar between the LFD group and the control group, while no data were provided. The other two studies did not report nutrient data.

### 3.2. Outcomes Combining Gut Microbiota and Mental Health

No studies in this review included all three topics or the two specific topics of gut microbiota and sleep.

Two studies identified gut microbiota and mental health in their results. Azpiroz et al. (2017) [92] assessed the effects of 5 g/d short-chain fructooligosaccharides (scFOS) or placebo for 4 weeks in IBS patients classified using the Rome III criteria. In contrast, Silk et al. (2009) [93] conducted an RCT with a crossover design between three groups (two treatment and one placebo), introducing 3.5 g or 7 g of trans-galactooligosaccharide (GOS) in patients with Rome II positive IBS. All subjects in the above study had a 2-week baseline, then were randomised into three groups for the two 4-week interventions with a 2-week washout phase in between. All participants started on a 4-week placebo treatment and, following the 2-week washout, then proceeded on a 4-week treatment (3.5 g GOS in Group I; 7 g GOS in Group II; 7 g placebo in Group III).

Stool samples were collected pre-and post-intervention for faecal microbiota analysis in both studies. qPCR was conducted in Azpiroz et al.’s research for describing the dominant taxonomic groups of the faecal microbiota. In contrast, Silk et al. used fluorescent in situ hybridisation to determine total bacterial counts and individual groups of faecal bacteria. A key finding in both studies despite prebiotics supplement (scFOS and GOS) was an increased abundance of faecal bifidobacteria. Additionally, the 4-week administrations of 3.5 g and 7 g GOS resulted in a significant increase in the relative proportion of *Bifidobacterium* spp compared to the placebo and the increase higher in the 7 g GOS group. In the scFOS study, the increase in bifidobacteria was significantly increased at the end of the study within the scFOS group, but this difference was not seen between the scFOS and placebo groups. Both studies assessed mental health using a validated questionnaire (Hospital Anxiety Depression Scale (HADS)), where both prebiotics of 5 g/day scFOS and 7 g/day GOS resulted in a significant reduction in HADS anxiety scores.

### 3.3. Outcomes Combining Sleep and Mental Health

Three studies included sleep and mental health as primary outcomes to determine the effectiveness of LFD as a treatment, all of which were delivered via qualified dietitians/nutritionists. Eswaran et al. [91] compared a 4-week LFD to the mNICE diet (modified diet recommended by the National Institute for Health and Care Excellence) [33]. In contrast, Bellini et al. [95] compared before and after an 8-week LFD in a single-arm study, while Kortlever et al. [94] provided participants with a dietitian’s consultation of LFD at baseline with follow-up at 6 and 26 weeks. The Rome III criteria were used to diagnose patients in all three studies.

No objective sleep measures were used in the five studies. Sleep was measured using subjective self-report questionnaires, including daily sleep quality ratings and pre/post modified sleep questionnaires, Pittsburgh Sleep Quality Index (PSQI), and Karolinska Sleep Questionnaire, respectively. For mental health assessment, HADS was applied in both Eswaran’s and Bellini’s studies [91,95], while Kortlever et al. assessed mental health using the State-Trait Personality Inventory (psychological indices concerning depression and anxiety). Notably, anxiety improved in all three of these studies. Eswaran et al. reported that sleep, anxiety, and depression all improved in the LFD group compared to the baseline, whereas only anxiety improved in the mNICE group. After 8-week of LFD, Bellini et al. identified an improvement in anxiety but not depression using HADS. They also did not record any improvements in sleep quality using PSQI. Kortlever et al.’s prospective observational study found improvement in anxiety after both 6 weeks and 26 weeks and in depression scores at 26 weeks, but no change was detected in sleep.

## 4. Discussion

This scoping review aims to survey the current evidence, including two out of the three primary outcomes of interest, namely gut microbiota, sleep, and mental health in IBS populations with dietary intervention. According to our results, none of the fibre-related interventional studies investigated all three outcomes and gut microbiota and sleep in combination. Five research studies were included, with two studies examining the relationship of the gut microbiota and mental health and three studies including sleep and mental health in their analysis. As scientific discovery covering gut microbiota and linking it to brain and behaviour is still being established, the association between sleep and diet [83], as well as mental health and gut microbiota [74,97], are gradually becoming acknowledged. Since diet is regarded as the main determiner of human gut microbiota [98], the relationship between diet, sleep, and mental health requires further consideration of gut microbiota in order to close the knowledge gap.

In all five studies included in this scoping review, only improved self-reported anxiety for IBS volunteers when following a fibre-related intervention was shared across all studies. Many animal studies have supported similar findings, where a prebiotic combination of GOS and polydextrose (PDX) has been shown to increase *Lactobacillus* spp. and *Bifidobacterium* spp. in rat faeces, attenuating anxiety-like behaviours [99]. Similarly, early-life supplementation of GOS and PDX can distinctly reduce stress-induced behaviours in mice [100]. Human studies are also attempting to determine the mechanism and interrelation between anxiety-depressive states, gut microbiota, and IBS itself, which has been associated with alterations in stress-induced inflammation, gut-oriented hormones such as serotonin and peptide YY (PYY), as well as microbial-mediated metabolites such as SCFA [75,101]. It is still unknown as to whether there exists a certain gut microbial profile that is linked to positive or negative mental health [74] and whether the human gut microbiota acts as a communicative hub [74,76] or acts as an aetiologic origin of disordered mental health [77,78]. Nonetheless, gut microbiota modulation remains a valuable strategy for people with mental health issues [102]. Encouraging improvement has been achieved simply via dietary adaptations, adding prebiotics and/or probiotics in appropriate amounts [103,104], where long-term sustained benefits undoubtedly require considerations and adjustments in the overall dietary pattern.

### 4.1. Dietary Fibre Intake in IBS

Only two studies [93,95] in this scoping review reported on baseline dietary fibre intake (Table 2), where participants in both failed to meet the dietary reference value regardless of national dietary recommendations. Similarly, the findings from Staudacher et al. [35] demonstrate that many IBS individuals did not adhere to the recommended fibre intake. Notably, this trend is not unique to IBS populations as it commonly occurs in the general population. The mean fibre intake among U.K. adults (19–65 years) was 19 g/day based on reports in 2015 and 2018/2019 [105], where only 9% of adults consumed the daily recommended amount of fibre (30 g) [106]. Another U.K. research using data of supermarket sales transaction of the whole year of 2016 (*n* = 299,260) found out that the average fibre intake was 16 g/day, where the most, 21 g/day, was seen in people with “fruity” dietary pattern (defined as 7 of the top 10 purchased items being types of fruit) [107]. Among U.S. adults (>19 years of age), dietary fibre intake in 2009 was 13.7 g/day in females and 17.6 g/day in males, with only 6% and <3% meeting the AI recommendation, respectively [108]. In the countries of the Eastern Mediterranean region, the average daily intake of fibre was 21.8 g/day (95% confidence interval: 19.6–24.1), according to the finding of a recent meta-analysis based on 43 studies (*n* = 72,534 subjects) published in recent nine years [109]. Accordingly, low dietary fibre intake in the general population appears to be a global issue.

Among IBS populations, certain dietary fibres [86] have been listed as a tool for overall symptom improvement, depending on physicochemical characteristics, including viscosity, solubility, and fermentability. A narrative review of meta-analyses published in March 2020 [110] found that four out of five meta-analyses suggested that fibre supplementation could provide significant clinical improvement via the Global Assessment of IBS Symptoms evaluation. This is also reflected in the recent Japanese IBS treatment guidelines suggesting bulking polymers or dietary fibre as an effective IBS treatment ranked as Level A evidence—strong recommendation [111], although no specific amount was specified.

In summary, what is currently lacking is probably not the exploration of optimal quantity of fibre intake, but rather the exploration of how to increase fibre intake in IBS individuals for symptoms-attenuation or non-exacerbation of symptoms.

### 4.2. Dietary Fibre Administration for People with IBS

With their relatively safe and inexpensive characteristics, dietary fibres can be widely applied to improve symptoms for people with IBS [87]. However, it may be difficult for IBS individuals to find replacement foods that are rich in fibre and potentially low in FODMAP [112] or ones they can tolerate well in order to avoid symptom attenuation. Therefore, it would be favourable and cost-effective for patients if they can manage the disorder via a combination of certain isolated fibres that optimises their gut microenvironment and gut function in place of whole foods. Clinically, sufficient guidance and education are also required so that patients can optimise their daily fibre intake without symptoms exacerbation, as well as can ensure the overall diet quality, overcome the potential challenges and minimise the possible detrimental effects of applied LFD [113].

There is evidence in the literature that a combination of isolated fibres, as a diet supplement, may be an option for IBS patients to improve their gut health. In IBS patients, short-chained carbohydrates resistant to digestion in the small intestine are rapidly fermentable in the proximal colon, where they can aggravate gastro-symptoms. However, in animal studies, it has been demonstrated that the rapid fermentation of RS can be mediated by moving the fermentation down towards the distal colon using psyllium [114]. Morita et al. [114] demonstrated in rats that psyllium can shift the RS2 (high amylose maize starch) fermentation further distally, by which butyrate production appears increased both in the distal colonic region and faeces. This is due to psyllium’s slow fermentability, strong gel-forming, and water-holding capacity, which traps RS granules and protects against proximal colonic fermentation, thereby delivering RS to the distal colon [114]. Similarly, the effects of RS-fermentation delay and higher butyrate production were also observed in a pig study using wheat bran and RS [115]. This has also been demonstrated in healthy volunteers where the supplementation of wheat bran combined with RS [116,117] can modulate gut microbiota, increasing butyrate and butyrate-producing bacteria. Interestingly, in contrast to wheat bran, psyllium is well tolerated by IBS subjects and has been deemed as an effective non-pharmaceutical management tool by authoritative bodies to improve overall symptoms, particularly for IBS-C patients [19,118]. The favourable effects of the gut-oriented metabolites relevant to the administration of dietary fibre in IBS individuals require further exploration.

Accordingly, it appears promising that IBS management can be enhanced with fibre co-administration that maximise the effects of dysbiosis normalisation while improving symptoms [42]. This may be a future direction for IBS-related research. In this regard, the therapeutic function and tolerability also need to be taken into consideration.

### 4.3. Evolution of Diagnosis Guideline and Potential Impacts

In accordance with the results of this scoping review, four of the five included studies used the Rome III criteria, while only one remaining study used Rome II. Therefore, IBS-relevant research using the newest Rome IV criteria is required. While the Rome IV version was published 10 years after its predecessor in 2016, as a result of its stricter criteria, it may prove an obstacle for researchers in recruiting patients with IBS. According to a meta-analysis published in 2020, the global prevalence of IBS dropped from 9.2% to 3.8%, which would mean that part of existing IBS populations diagnosed by Rome III would technically be reclassified, based on the Rome IV, as “no bowel disorder or unspecified functional bowel disorders” [119]. This has led the Vice-Chair of Administration of the University of Tennessee Health Science Center in 2020 to raise a query as to whether the Rome criteria is a sound diagnosis for GI disorders [120].

Nonetheless, whether one in 11 (according to the Rome III criteria) or one in 26 people (according to the Rome IV criteria) are classified as suffering from IBS [119], these people still require optimal solutions and support. Based on patient reports of real-life experiences, IBS patients have struggled with various physical (i.e., GI symptoms and fatigue), psychological (i.e., depression and anxiety), and social (i.e., avoiding activities and long-distance travel, limited food choices) consequences [121]. Hence, these factors further function as stressors and triggers, that together with their precursors, constitute a vicious cycle.

### 4.4. Sleep Hierarchical Assessment Methods

The wide availability and application of wearable sleep monitoring technologies, such as sleep tracking devices, have exhibited high performance in sleep-wake detection [122]. These devices are home-based and cost-effective have enabled an increase in sleep-related research [123,124], facilitating multi-disciplinary research that provides insight into sleep changes based on variable study settings.

**Figure 2:**
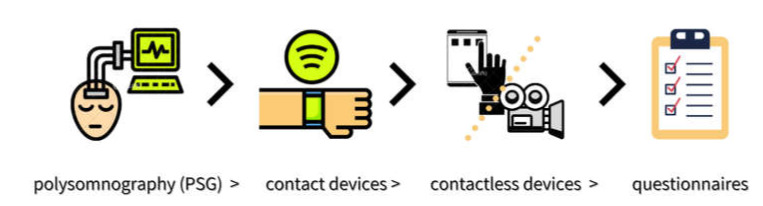
A relative hierarchy in order of sleep assessment methods.

With regards to sleep as an outcome measure in this scoping review, only the LFD group in Eswaran’s research showed an improvement. However, no objective sleep measures were applied in any of the studies included. Possible explanations for the lack of observable change could be due to either changes in sleep not occurring, changes not being identified, or studies being underpowered to detect a change. These factors cannot be neglected. Every method for assessing sleep has potential strengths and shortcomings, where there exists a relative hierarchy in order of accuracy, including: polysomnography (PSG)-gold standard > contact devices > contactless devices > questionnaires [125] (Figure 2). In relative terms, due to subjective perception about one’s own quality of sleep and memory bias, it is likely that data obtained in sleep questionnaires can be biased and inconsistent as compared with data from validated devices [125]. Therefore, in future research, it would be beneficial to combine subjective and objective methods of data collection in order to obtain more reliable and accurate results in IBS-related studies.

Diet-derived sleep improvement has also been previously documented [126], and healthy adults’ sleep can be negatively impacted when people shift their diet towards low fibre and high saturated fat and sugar intake [127]. Animal studies have shown that dietary prebiotics [128] and their metabolite, butyrate [68], can improve sleep.

### 4.5. Limitations

The scoping review extracted the outcomes on the three topics of interest (gut microbiota, sleep, and mental health) rather than all outcomes in the included studies. As diet is the main determinant of human gut microbiota, this review only targeted fibre-related interventions as one of the inclusive criteria, where other treatments such as psychotherapy, including gut-focused hypnotherapy, cognitive behaviour therapy (CBT), and mindfulness were not assessed. Finally, the review limited the subjects studied to IBS adults only, where children and elderly participants and animal studies were not included in the scoping exercise.

### 4.6. Future Research Recommendations

With regard to sleep assessment specifically, a high level of accuracy and reliability of sleep measures, combining objective and subjective methods if applicable, is essential to ensure the quality of data collected. Additionally, studies with psychotherapy treatments or behavioural therapies for IBS could also be mapped in future reviews targeting the three themes as outcomes for a better understanding of the link, if any exists, between gut microbiota and sleep and mental health. Notably, effects resulted from diet-related intervention in gut microbiota and sleep require considerations in IBS subtypes, habitual diet, and the baseline (pre-interventional) gut microbial phenotype [74]. This is related to host interactive influence, where different microbial compositions may result in similar functions and vice versa [74]; while the intertwined association between host habitual diet and gut microbiota [45,98], as well as sleep and gut microbes [71,73,129], has not been fully identified.

During the searching process for this review, three study protocols were identified that targeted all three themes together [130], with one adding structural magnetic resonance imaging (MRI) of the human brain [131] and another creating a massive prospective cohort of people with IBS, inflammatory bowel disease, and healthy individuals combing genetic, microbiome, and metabolomic profiles [132]. All these scientific advancements are promising, potentially introducing a new era of “microbiome literacy” from “food literacy”.

For IBS non-pharmaceutical management, it is worthwhile to explore dual or multi-administration of dietary fibres that are well tolerated, therapeutically functional, and capable of promoting the entire or local intestinal microbial environment. Even though a one-size-fits-all treatment for IBS individuals does not exist, individualising/personalising dietary plans can be an optimal and economically feasible solution for this population. It is likely that the gut microbiome is the main determinant of the diet-health relationships [133]. Therefore, further exploration of the “pieces of the puzzle” around gut microbiota, sleep, mental health, and habitual diet in IBS is still required.

## 5. Conclusions

This scoping review has highlighted the lack of IBS-relevant research targeting the three themes of gut microbiota, sleep, and mental health as outcomes when administering a dietary intervention. Future work should continue to focus on diet-related interventions, either for alterations in a whole dietary pattern or for specific components of the diet or supplementation as needed to manage IBS symptoms with emphasis on improving the dysbiotic gut environment that can further improve sleep and mental health outcomes among IBS populations. Additionally, the application of objective sleep assessment methods is required to detect sleep change with more accuracy and less bias.

## Figures and Tables

**Table 1:** Search keywords.

Concept	IBS	Fibre	Gut Microbiota	Sleep	Mental Health
Keywords used in the search	IBS OR irritable bowel syndrome	diet * OR diet therapy OR diet * fibre OR diet * fibre OR fib * supplement OR fermentable carbohydrate OR FODMAP OR low-FODMAP OR low-FODMAP diet OR prebiotic	intestinal flora OR Gut microbio * OR gastrointestinal microbiome OR microbio * OR gut flora OR dysbiosis	sleep * OR insomnia OR sleep disorder * OR sleep problem * OR sleep deprivation OR sleep fragmentation * OR sleep disturbance OR sleep disruption OR sleep loss OR sleepless	mental * OR mental health

Truncation (*) was applied in the prosses of databases searching.

**Table 2:** Summary and characteristics of included studies.

Title	First Author, Country and Year	Type of Study	Participants			Dietary Intervention Approach	Intervention and Group Setting	Gut Microbiota Outcomes	Sleep (Subjective Questionnaire)	Mental Health Questionnaire	Outcomes in Sleep and Mental Health	Adverse Effects
			*N* Mean Age (Range)	Rome Criteria and IBS Subtype	Baseline Fibre Intake (g/day)							
A diet low in fermentable oligo-, di-, and monosaccharides and polyols improves quality of life and reduces activity impairment in patients with irritable bowel syndrome and diarrhoea	Eswaran, S. USA 2017	RCT	*N* = 92; 42.6 (19–75)	III; IBS-D (female)		Dietitian consultations on the allocated diet	4 week diet interventions Low-FODMAP diet Group *n* = 50 mNICE * Group *n* = 42		Daily sleep quality rating and pre/post modified sleep questionnaire	HADS	HADS anxiety and depression and sleep all improved on Low-FODMAP diet group compared with baseline. Anxiety improved in mNICE group	NA
Effects of scFOS on the composition of faecal microbiota and anxiety in patients with irritable bowel syndrome: a randomised, double blind, placebo-controlled study	Azpiroz, F. Spain and France2017	RCT	*N* = 79; 41/42.4 ** (18–60)	III; IBS-D IBS-C IBS-M IBS-U	<20 g/day as required	Fibre supplementation	4 week 5 g/day scFOS *n* = 41 placebo *n* = 38	scFOS increased faecal bifidobacteria		HAD	Contrary with placebo, scFOS significantly reduced anxiety scores	*n* = 18, scFOS *n* = 21, placebo Symptoms were not reported
Clinical trial: the effects of a trans-galactooligosaccharide prebiotic on faecal microbiota and symptoms in irritable bowel syndrome	Silk, D.B.A. U.K. 2019	Crossover RCT	*N* = 44; 54 (20–79)	II; IBS-D IBS-C IBS-A	Group I: 13.1 ± 4.06 Group II: 9.4 ± 3.46 Group III: 10.9 ± 5.04	Fibre supplementation	2 week baseline -> 4 week treatment -> 2 week washout -> 4 week treatment Group I *n* = 16: placebo 7 g/day --> GOS 3.5 g; Group II *n* = 14: placebo 7 g/day --> GOS 7 g; Group III *n* = 14: placebo 7 g/day -> placebo 7 day/g	GOS enhanced faecal bifidobacteria		HAD	GOS significantly improved anxiety scores compared to placebo treatment	*n* = 3 (moderate diarrhoea, *n* = 1; mild nausea = 2)
Bioelectrical impedance vector analysis in patients with irritable bowel syndrome on a low-FODMAP diet: A pilot study	Bellini, M. Italy 2017	A pilot study, single arm	*N* = 26; 46.2 (18–65)	III; IBS-D IBS-C IBS-M	20.5 ± 10.7	Nutritionist instruction	8 week low-FODMAP diet		PSQI	HADS	HADS anxiety improved, PSQI and HADS depression did not improve	NA
Low-FODMAP diet is associated with improved quality of life in IBS patients—A prospective observational study	Tim, L.; Kortlever, NZ and AU 2019	A prospective observational study	*N* = 101; 41.9 (16–75)	III; IBS-D IBS-C IBS-M IBS-U	\	Dietitian consultations at baseline and follow-up	dietitian consultation of low-FODMAP diet at baseline follow-up at week 6 (*n* = 70) and week 26 (*n* = 51)		Karolinska Sleep Questionnaire	State-Trait Personality Inventory ***	Anxiety improved at T6 and T26; Depression improved at T26; sleep did not improve	NA

Abbreviations: N: sample size. NA: not available. IBS-D: diarrhoea dominant IBS; IBS-C: constipation dominant IBS; IBS-A: alternating IBS; IBS-U: unclassified IBS; IBS-M: mixed bowel habits IBS. HAD/HADS: Hospital Anxiety and Depression Scale. scFOS: short-chain fructooligosaccharides. mNICE: modified diet recommended by the National Institute for Health and Care Excellence. PSQI: Pittsburgh Sleep Quality Index. * The mNICE group was instructed to eat small frequent meals and avoid trigger foods, excess alcohol, and caffeine. ** The mean ages reported separately in two groups (41 in scFOS group, 42.4 in placebo group). *** State-Trait Personality Inventory: psychological indices concerning depression and anxiety.

## Data Availability

Data sharing not applicable.
